# Manual choice reaction times in the rate-domain

**DOI:** 10.3389/fnhum.2014.00418

**Published:** 2014-06-10

**Authors:** Christopher M. Harris, Jonathan Waddington, Valerio Biscione, Sean Manzi

**Affiliations:** ^1^Centre for Robotics and Neural Systems and Cognition Institute, Plymouth UniversityPlymouth, UK; ^2^School of Psychology, Plymouth UniversityPlymouth, UK; ^3^The WESC FoundationExeter, UK; ^4^School of Psychology, University of LincolnLincoln, UK

**Keywords:** reaction times, latency, reciprocal Normal, autoregressive integrated moving average (ARIMA), speed-accuracy trade-off, Pieron's law, optimality

## Abstract

Over the last 150 years, human manual reaction times (RTs) have been recorded countless times. Yet, our understanding of them remains remarkably poor. RTs are highly variable with positively skewed frequency distributions, often modeled as an inverse Gaussian distribution reflecting a stochastic rise to threshold (diffusion process). However, latency distributions of saccades are very close to the reciprocal Normal, suggesting that “rate” (reciprocal RT) may be the more fundamental variable. We explored whether this phenomenon extends to choice manual RTs. We recorded two-alternative choice RTs from 24 subjects, each with 4 blocks of 200 trials with two task difficulties (easy vs. difficult discrimination) and two instruction sets (urgent vs. accurate). We found that rate distributions were, indeed, very close to Normal, shifting to lower rates with increasing difficulty and accuracy, and for some blocks they appeared to become left-truncated, but still close to Normal. Using autoregressive techniques, we found temporal sequential dependencies for lags of at least 3. We identified a transient and steady-state component in each block. Because rates were Normal, we were able to estimate autoregressive weights using the Box-Jenkins technique, and convert to a moving average model using z-transforms to show explicit dependence on stimulus input. We also found a spatial sequential dependence for the previous 3 lags depending on whether the laterality of previous trials was repeated or alternated. This was partially dissociated from temporal dependency as it only occurred in the easy tasks. We conclude that 2-alternative choice manual RT distributions are close to reciprocal Normal and not the inverse Gaussian. This is not consistent with stochastic rise to threshold models, and we propose a simple optimality model in which reward is maximized to yield to an optimal rate, and hence an optimal time to respond. We discuss how it might be implemented.

## Introduction

Reaction times (response times, latency) (RTs) have been measured and discussed innumerable times since their first measurements in the mid-19th century by von Helmholtz (1850) and Donders (1969). RT experiments are so commonplace that they have become a standard paradigm for measuring behavioral responses, often with scant regard to any underlying process. However, the mechanisms behind RTs are complex and poorly understood. A common view is that RTs reflect processing in the time-domain, where RTs are the sum of independent sequential processes including conduction delays, decision-making processes, and motor responses. We question this very fundamental assumption and consider responses in the rate-domain, where rate is defined as the reciprocal of RT.

One of the most perplexing aspects of RTs is their extreme variability from one trial to the next with some very long RTs, even when the same stimulus is repeated and subjects are instructed to respond as quickly as possible. As exemplified by the saccadic system, why does it take hundreds of milliseconds to decide to make a saccade, when the saccade itself only takes a few tens of milliseconds to execute (Carpenter, 1981)? Moreover, if we accept that point-to-point movements, such as saccades and arm reaching are time-optimal (Harris and Wolpert, 1998), should we not expect the RT also to be optimized? One is then led to wonder how such long response times could be optimal.

### Drift diffusion models (DDM)

**Figure 1:**
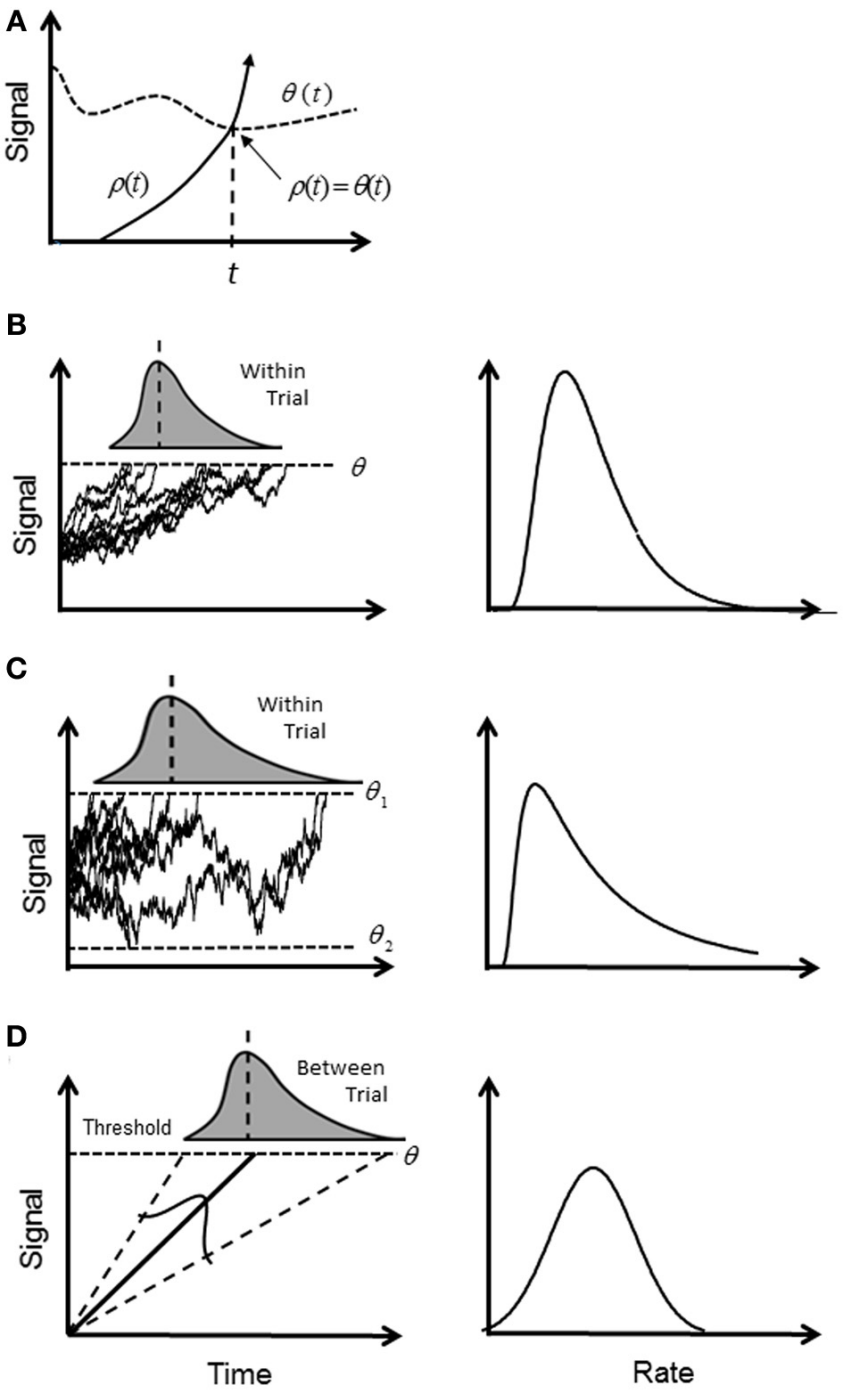
**Illustration of accumulator models: (A) general first-passage scheme where a triggered event occurs when a signal ρ (*t*) first crosses the trigger level θ (*t*)**. Note that crossing is a solution to the equation ρ (*t*) = θ (*t*); **(B)** in the diffusion model ρ (*t*) increases stochastically and triggers a response when it reaches a constant θ (*t*), or “threshold.” The signal is assumed to be a Wiener process, and the first passage time is a within-trial random variable (shaded curve) with an inverse Gaussian distribution. In the rate domain (right column) the rate distribution remains positively skewed; **(C)** the diffusion model for two boundaries, where boundary θ _1_ (*t*) determines correct responses, and boundary θ _2_ (*t*) determines error responses. In the rate domain, rate of correct responses remains positively skewed. **(D)** In the deterministic model, ρ (*t*) increases linearly and deterministically until the threshold is reached. It is assumed that the slope of rise is a between-trial Normal random variable and gives rise to a reciprocal Normal distribution. In the rate domain, rate is distributed with a truncated Normal distribution.

The most popular explanation for the variability of RTs has revolved around the putative mechanism of an accumulator or “rise to threshold” model. A signal, ρ (*t*), increases (accumulates) in time until it crosses a boundary (“trigger level” or “decision threshold”), θ (*t*), whereupon the response is initiated (first-passage time; Figure 1A). Typically, ρ (*t*) is assumed to be a stochastic signal reflecting the accumulation of “information” for or against an alternative until a predetermined level of confidence is reached represented by a constant θ (*t*) (Ratcliff, 1978) (Figure 1B). A simple reaction time is modeled by a single boundary, and a two-alternative choice task is modeled by two boundaries. A RT is then first-passage time for one of the alternatives plus any other “non-decision” time such as sensorimotor delays (e.g., Ratcliff and Rouder, 1998; Ratcliff et al., 1999).

Typically, ρ(*t*) is assumed to drift with a constant mean rate but is instantaneously perturbed by a stationary Normal white noise process (Wiener process), so that within a given trial and with one boundary, the time of crossing the threshold is a random variable with an inverse Gaussian distribution (Schrodinger, 1915; Wald, 1945). With two boundaries, the first passage time for one boundary indicates the decision time for a correct response, and an error response for the other boundary; their probability density functions (pdf's) are computed numerically (Ratcliff, 1978; Ratcliff and Tuerlinckx, 2002) (see Table 1 for pdf's). For an easy choice task (i.e., high drift rate toward the “correct” boundary), the pdf will approach the inverse Gaussian distribution as error rate become negligible. Although, there are numerous variations on this theme (e.g., Ratcliff and Rouder, 1998, 2000; Smith and Ratcliff, 2004; Bogacz et al., 2006; Ratcliff and Starns, 2013), they share the same basic stochastic rise to threshold decision-making process in the time-domain. It has been recently shown how the pure diffusion process (without variability across trials) has an exact equivalent in terms of Bayesian inference (Bitzer et al., 2014). As shown by Bogacz et al. (2006), the DDM is optimal in the sense that for a given boundary (decision accuracy) the decision is made in minimal time.

**Table 1:** **Left column: mathematical expressions of the probability density functions (pdf's) for RTs for a single boundary diffusion model, two boundary diffusion model, and the reciprocal Normal**.

**Time-domain**	**Rate-domain**
Inverse Gaussian	Reciprocal inverse Gaussian
$IG(\mu ,\sigma^{2})={\left(\frac{\mu^{3}}{2\pi \sigma^{2}\chi^{3}}\right)}^{\frac{1}{2}}\exp\left[\frac{-\mu {\left(\chi -\mu \right)}^{2}}{2\sigma^{2}\chi }\right]$	$recIG(\mu ,\sigma^{2})={\left(\frac{\mu^{3}}{2\pi \sigma^{2}\chi }\right)}^{\frac{1}{2}}\exp\left[\frac{2\mu^{2}-\mu /\chi -\chi \mu^{3}}{2\sigma^{2}}\right]$
First passage time distribution for the boundary of the two boundaries *a* and *b* for the pure DDM with diffusion constant, *s*	Reciprocal first passage time distribution boundary a of the two boundaries a DDM
$\begin{array}{l} B(\xi ,a,b,z)=\frac{\pi s^{2}}{{\left(a-b\right)}^{2}}\exp\left[\frac{\xi (a-z)}{s^{2}}-\frac{\xi^{2}t}{2s^{2}}\right]\\ \sum_{k\;=\;1}^{\infty }k\exp\left[-\frac{k^{2}\pi^{2}s^{2}t}{2{\left(a-b\right)}^{2}}\right]\sin\left[\frac{k\pi (a-z)}{a-b}\right] \end{array}$	$\begin{array}{l} recB(\xi ,a,b,z)=\frac{\pi s^{2}}{t^{2}{\left(a-b\right)}^{2}}\exp\left[\frac{\xi (a-z)}{s^{2}}-\frac{\xi^{2}}{2ts^{2}}\right]\\ \sum_{k\;=\;1}^{\infty }k\exp\left[-\frac{k^{2}\pi^{2}s^{2}}{2t{\left(a-b\right)}^{2}}\right]\sin\left[\frac{k\pi (a-z)}{a-b}\right] \end{array}$
see Ratcliff and Smith (2004)	
Reciprocal truncated Normal	Truncated Normal
$rectrN(\mu ,\sigma )=\frac{\exp\left[-\frac{{\left(1/t-\mu \right)}^{2}}{2\sigma^{2}}\right]}{\sqrt{2\pi }\left(1-\phi \left(-\frac{\mu }{\sigma }\right)\right)\sigma t^{2}}$	$trN(\mu ,\sigma )=\frac{\exp\left[-\frac{{\left(t-\mu \right)}^{2}}{2\sigma^{2}}\right]}{\sqrt{2\pi }\left(1-\varphi \left(-\frac{\mu }{\sigma }\right)\right)\sigma }$

Φ = Normal cdf; ξ = drift rate; a = upper boundary; b = lower boundary; z = starting point. Right column: equivalent pdf's in the rate (reciprocal RT) domain. See Harris and Waddington (2012) for the mathematical relationship between the two domains.

Ratcliff (1978) also allowed the mean drift rate to fluctuate between trials with a Normal distribution to reflect “stimulus encoding” variability. This version has often been called the extended DDM, which also includes variability in the starting point of drift, and variability in the non-decision component (Ratcliff and Tuerlinckx, 2002). The extended DDM has been used to describe simple RT experiment (Ratcliff and van Dongen, 2011) and choice RT (Ratcliff, 1978; Hanes and Schall, 1996; Ratcliff and Rouder, 1998; Schall, 2001; Shadlen and Newsome, 2001; Ratcliff et al., 2003, 2004; Smith and Ratcliff, 2004; Wagenmakers et al., 2004; Ratcliff and McKoon, 2008; Roxin and Ledberg, 2008).

Although the multi-parameter extended DDM is claimed to fit observations, a serious problem has emerged from the eye movement literature, when we consider the distribution of the reciprocal of RTs, which we call “rate.”

### The reciprocal normal distribution

Investigations into the timing of saccades for supra-threshold stimuli have shown that the frequency distribution of simple RTs (latency) is close to the reciprocal Normal distribution; that is, rate has a near-Normal distribution. Small deviations from true Normal are observed in the tails, but probit plots are typically linear between at least the 5th and 95th centiles (Carpenter, 1981). The reciprocal Normal is not known to be a first-passage distribution for a constant threshold, and is easily distinguished from the inverse Gaussian or the two-boundary pdf. Carpenter has proposed the LATER model in which the rise to threshold is linear and deterministic, but the slope of rise varies from trial to trial with a Normal distribution (Carpenter, 1981; Carpenter and Williams, 1995; Reddi and Carpenter, 2000) (Figure 1D). If Carpenter's findings can be generalized beyond saccades, they are equivalent to the extended DDM without fluctuation in the rise of ρ (*t*) (i.e., no diffusion) and with only one threshold. There is an obvious difficulty in how to explain a deterministic rise to threshold based on a Bayesian update rule, which is inherently stochastic. Moreover, if the rise is deterministic then the time to reach threshold is known at the outset, and any competition among alternatives can be resolved very quickly—so why wait?

The reciprocal Normal is a bimodal distribution with positive and negative modes. In the time-domain this would imply very large negative RTs, which would require the response to occur long before the stimulus onset and violate causality. Therefore, we need to consider the reciprocal truncated Normal distribution (**rectrN**), (where the Normal rate distribution is left truncated at or near zero; see Harris and Waddington, 2012). The question is what happens at or near zero rate? For easy tasks where RTs are low, the probability of rate reaching zero (i.e., RT approaching infinity) is negligible and the problem might be dismissed as a mathematical nuance. However, for difficult tasks, the probability becomes significant, as we have shown (Harris and Waddington, 2012). A departure from the reciprocal Normal has been reported for saccade latency to very dim targets, but this has been modeled instead as an inverse Gaussian based on a diffusion process (Carpenter et al., 2009). Clarification is needed on what happens when rates are low.

It has long been known that sequential effects occur in manual choice RTs (Hyman, 1953). In sequences of 2-alternative choice RT experiments, RTs may be correlated with the previous trial (first-order) and also earlier trials (high-order). Moreover, this sequential dependency seems to be a function of whether a stimulus is repeated or alternated (Kirby, 1976; Jentzsch and Sommer, 2002). Sequential dependencies cannot be explained by within-trial noise processes, such as the DDM, unless there are between-trial parameter changes (changes in drift rate or threshold values). If we assume a linear dependence on history (autoregressive model) in the rate-domain, then it could in principle lead to convergence onto the Normal distribution via the central limit theorem.

### The rate-domain

It is important, therefore, to identify RT distributions, but this is a non-trivial problem. It is difficult to distinguish among highly skewed distributions in the time-domain. The method of moments is infeasible due to poor convergence (the reciprocal Normal has no finite moments; Harris and Waddington, 2012). Maximum likelihood estimation of parameters requires vast amounts of data to distinguish between models (Waddington and Harris, 2012). There is also the problem of under-sampling at extreme values (Harris and Waddington, 2012) which is further exacerbated by the tendency of many investigators to discard “outliers.” It is easier in the rate-domain, although large data sets are still needed. Distributions that are less skewed than the reciprocal Normal (such as the inverse Gaussian) remain positively skewed in the rate-domain, whereas the reciprocal Normal does not. Surprisingly, there have only been a few published examples of manual reaction times in the rate-domain (Carpenter, 1999; Harris and Waddington, 2012), and it is conceivable that saccades are somehow “special.” For example, express saccades do not appear to have an equivalent in manual tasks. Another important issue is lack of stationarity, where the mean and variance (and higher moments for non-Normal distributions) change over time. Non-stationarity of the mean is particularly troublesome because it smears out the observed distribution making the RT distribution more platykurtic and heavy-tailed. Non-stationarity is more likely in long recording sessions, as subjects become fatigued and bored by the repetitive nature of RT experiments. Using large sample sizes from prolonged recording sessions may be counterproductive.

When a probability density function (pdf) is known in one domain, the pdf in the reciprocal domain can easily be found. However, it is important to recognize this is not true for moments. For example, the mean of the rate distribution is not the reciprocal of the mean of the RT distribution (Harris and Waddington, 2012). Thus, it is not possible to infer parametric statistics of rate from RT statistics. Raw data are needed. Therefore, our goal in this study was to explore rate-domain analysis in a typical two-choice manual RT experiment. We imposed two tasks (instruction set) and two levels of stimulus difficulty (brightness difference) in order to explore the effects of truncation, and we used autoregression analysis and z-transforms to examine sequential dependency. To minimize problems of non-stationarity, we recorded only modest block sizes (200) from many subjects (24) and collapsed after standardization. We show that rate is indeed near-Normal and not the reciprocal of the inverse Gaussian. Sequential dependency is evident, but not the cause of the near-Normality. In the discussion we propose a rate model as an alternative to first-passage time models.

## Methods

### Reaction time recording

Subjects were 24 adults aged between 18 and 45 years old selected through the Plymouth University paid participant pool as an opportunity sample. Subjects were naïve to the experimental procedure. Based on self-report, all participants were required to have normal or corrected-to-normal vision with no known neurological conditions. This study received ethical approval from the local ethics committee.

Stimuli consisted of two solid colored rectangles of different luminances arranged horizontally and displayed on a computer monitor (Hanns-G HA191, 1280 × 1024, at 60 Hz). Both rectangles were displayed in the same green color in Red-Green-Blue (RGB) coordinates against a gray background of luminance 37.1 cd/m^2^. Each rectangle subtended a visual angle of 5.5 horizontal and 6.6° vertically, and the inner edges were separated horizontally by 9.6°. Viewing distance was 0.5 m. Subjects were instructed to respond to the side with brighter stimulus by pressing the “z” or “2” key. In the easy task (E), rectangle luminances were 37.6 and 131.6 cd/m^2^, and in the difficult task (D), they were 37.6 and 37.8 cd/m^2^. Calibration was made with a Konica Minolta LS-100 luminance meter. All luminances and ambient room lighting were held constant for all subjects. The luminances in the (E) and (D) tasks were chosen to yield low and high error rates of 1% and 24% for these tasks respectively based on a pilot study. Two task instructions were used and displayed at the beginning of a block. In the “Urgent” (U) task, the instruction was to “respond as fast as possible,” and in the “Accurate” (A) task, to “respond as accurately as possible.” Each subject was presented with 4 blocks of 200 trials each. Within a block each trial consisted of the same combination of stimulus and task, either AE, AD, UE, or UD. There were 24 different permutations of blocks, and the order was balanced such that each of the 24 subjects had a unique order. We refer to the “easy” tasks as AE and UE, and the “difficult” tasks as AD and UD.

On each trial the subject was prompted to press the space key to commence the trial and a cross appeared in the center of the screen for 500 ms. Subsequently, the two rectangles appeared after a constant foreperiod of 500 ms. For choice reaction time experiments (unlike simple reaction time experiments), constant and variable foreperiods have similar effects (Bertelson and Tisseyre, 1968). We chose constant to avoid introducing additional variability into the decision process (see Discussion). Stimulus onset was also highly salient, even in the difficult tasks, due to the highly visible colored rectangles. The stimuli remained on screen until a response was made or until a time-out of 60 s occurred (see Harris and Waddington, 2012 for a discussion on the importance of a long time-out). For incorrect responses, feedback was provided in the form of a black cross, which remained on screen for 500 ms. A rest break occurred between blocks.

Reaction times (RTs) were measured from the onset of the stimulus presentation and recorded to the nearest millisecond. Rates were computed by taking the reciprocal RT. Taking reciprocals of integer RTs magnifies the effect of the quantization and can lead to artifactual “clumping” and “gaps” in the rate frequency histograms at high values of rate. We eliminated this by using a dithering technique, where we added a uniform floating point random number between −0.5 and +0.5 ms to each RT before taking the reciprocal (see Schuchman, 1964). This has no statistical effect in the time-domain. RTs less than 0.15 s (i.e., rate > 6.67 s^−1^) were considered anticipatory and not analyzed.

### Moments

Sample central moments (mean, standard deviation, skewness, and excess kurtosis) and medians were estimated for each block for RT and rate. Note that moments of RT and rate are not reciprocally related, but depend on the underlying parent distribution. However, median rate is the reciprocal of median RT (see Harris and Waddington, 2012).

We also estimated the mean and standard deviation in the rate-domain assuming the underlying distribution was Normal. The underlying mean and standard deviation of the Normal distribution will differ from the sample mean and standard deviation depending on how much of the underlying Normal distribution is truncated. We therefore obtained maximum likelihood estimates (MLEs) of the underlying Normal parameters from each dataset using the *mle.m* function. This function applied a simplex search algorithm to find the parameters that maximized the log likelihood of the probability density function:

$$f(x;\mu ,\sigma ,a)=|\begin{array}{cc} \frac{\varphi \left[\left(x-\mu \right)/\sigma \right]}{1-\Phi \left[\left(a-\mu \right)/\sigma \right]} & a\leq x<\infty \\ 0 & x<a \end{array}$$

where *x* is the observed rate, μ is the mean of the underlying (un-truncated) Normal distribution, σ is the standard deviation of the underlying distribution, *a* = 1/60 = 0.0167 s^−1^, φ is the standard Normal probability density function (pdf), and Φ is the standard Normal cumulative distribution function (cdf).

### Sequential analysis

The partial autocorrelation function (PACF) was computed using the *parcorr.m* Matlab function. The first 10 trials on each block were omitted to avoid contamination from initial transients. The coefficients for the first *m* = 20 lags were computed for each block and averaged across blocks. An autoregressive model (AR) was assumed to be of the form:

(1.1) $$r_{n}=a_{1}r_{n-1}+a_{2}r_{n-2}+\cdots +a_{m}r_{n-m}+u_{n}$$

where *r*_*i*_ is the response on the *i*th trial, *a*_*j*_, 1 < *j* < *m* are constant weights, and *u*_*i*_ is a stochastic input on the *i*th trial (negative indices were assumed to have zero weights). The autoregressive weights, *a*_*j*_ and input *u*_*i*_ are unknown and were estimated using the Box-Jenkins maximum likelihood procedure. We used the *estimate.m* function and an autoregressive integrated moving average (ARIMA) model with only an autoregressive polynomial (i.e., no non-seasonal differencing or moving average polynomials). We assumed the distributional form of *u*_*i*_ to be Normal with constant mean and variance.

An AR model can be converted to the equivalent moving average (MA) series using the standard z-transform method. The z-transforms Z(.) of (1.1) is

$$\begin{array}{l} R(z)=a_{1}z^{-1}R(z)+a_{2}z^{-2}R(z)+\cdots +a_{m}z^{-m}R(z)+U(z) \end{array}$$

where *R*(*z*) = *Z*(*r*), *U*(*z*) = *Z* (*s*). This can be viewed as a discrete time MA system with

*R*(*z*) = *B*(*z*)*U*(*z*)where the system response of order *m* is

$$\begin{array}{l} B(z)=\frac{1}{1-a_{1}z^{-1}-a_{2}z^{-2}-\cdots -a_{m}z^{-m}} \end{array}$$

To find *B*(*z*) we took a partial fraction expansion:

$$\begin{array}{l} B(z)=\sum_{i=k}^{m}\frac{\rho_{k}}{1-\lambda_{k}z^{-1}} \end{array}$$

where λ _*i*_ are the roots and ρ _*i*_ the residues Taking the inverse z-transform, we then have:

(1.2) $$\begin{array}{l} r_{n}=b_{0}u_{n}+b_{1}u_{n\;-1}+b_{2}u_{n\;-2}+\cdots \end{array}$$

where *u*_*k*_ is the stochastic input on trial *k* and independent of other trial inputs, *b*_0_ = 1, and *b*_*i*_ = $\sum_{i\;=\;1}^{m}\rho_{k}$ λ ^*i*^_*k*_, 1 ≤ *i* < ∞, and was computed in Matlab using the *roots.m* and *residue.m* functions. Note that (1.1) and (1.2) describe the same system, but (1.1) is a feedback description, and (1.2) is the feed-forward description. We chose 6 roots, as this encompassed the obviously larger PACF coefficients. The roots were all within the unit circle indicating stability and the existence of a steady-state.

### Steady-state transfer

From (1.2) we can relate the pdf of rate (output), *p*_*r*_ (*r*) to the pdf of the input where *u*_*i*_ are identical independent random variables with pdf *p*_*u*_ (*u*), *u* ≥ 0. From basic probability theory, (Papoullis and Pillai, 2002) the steady-state output pdf is given by the convolution sequence:

(1.3) $$\begin{array}{l} p_{r}(r)\text{​}=\text{​}\left[\frac{1}{\left|b_{0}\right|}p_{u}\left(\frac{u}{b_{0}}\right)\right]\text{​ }⊗\text{​ }\left[\frac{1}{\left|b_{1}\right|}p_{u}\left(\frac{u}{b_{1}}\right)\right]\text{ ​}⊗\text{ ​}\left[\frac{1}{\left|b_{2}\right|}p_{u}\left(\frac{u}{b_{2}}\right)\right]\text{ ​}⊗\text{ ​}\cdots \end{array}$$

where ⊗ is the convolution operator. If *p*_*u*_ (*u*) is Normally distributed then so is *p*_*r*_ (*r*). If *p*_*u*_ (*u*) is not Normal then *p*_*r*_ (*r*) may or may not converge to Normal depending on *p*_*u*_ (*u*) and the coefficients *b*_*i*_. We computed (1.3) numerically for the truncated Normal (see Results).

Consider the case where *p*_*u*_ (0) = *c* where *c* > 0 which corresponds to the case of truncation and when the RT distribution has no finite moments (see Harris and Waddington, 2012). For one term, we have *p*_*r*, 1_ (0) = *c* / |*b*_0_|. However, with two terms (one convolution) we have *p*_*r*, 2_ (*r*) = $\frac{1}{\left|b_{0}\right|\left|b_{1}\right|}$ ⨛ ^∞^_0_ *p*_*u*_ (*r* − $\frac{x}{\left|b_{0}\right|}$) *p*_*u*_ ($\frac{x}{\left|b_{1}\right|}$) *dx*. For *r* = 0 and *c* < ∞, *p*_*r*, 2_ (*r*) = 0. Similarly, for all terms we must have *p*_*r*_ (*r*) = 0, so that truncation is lost and the RT distribution will have a finite mean (but not necessarily higher moments).

## Results

**Figure 2:**
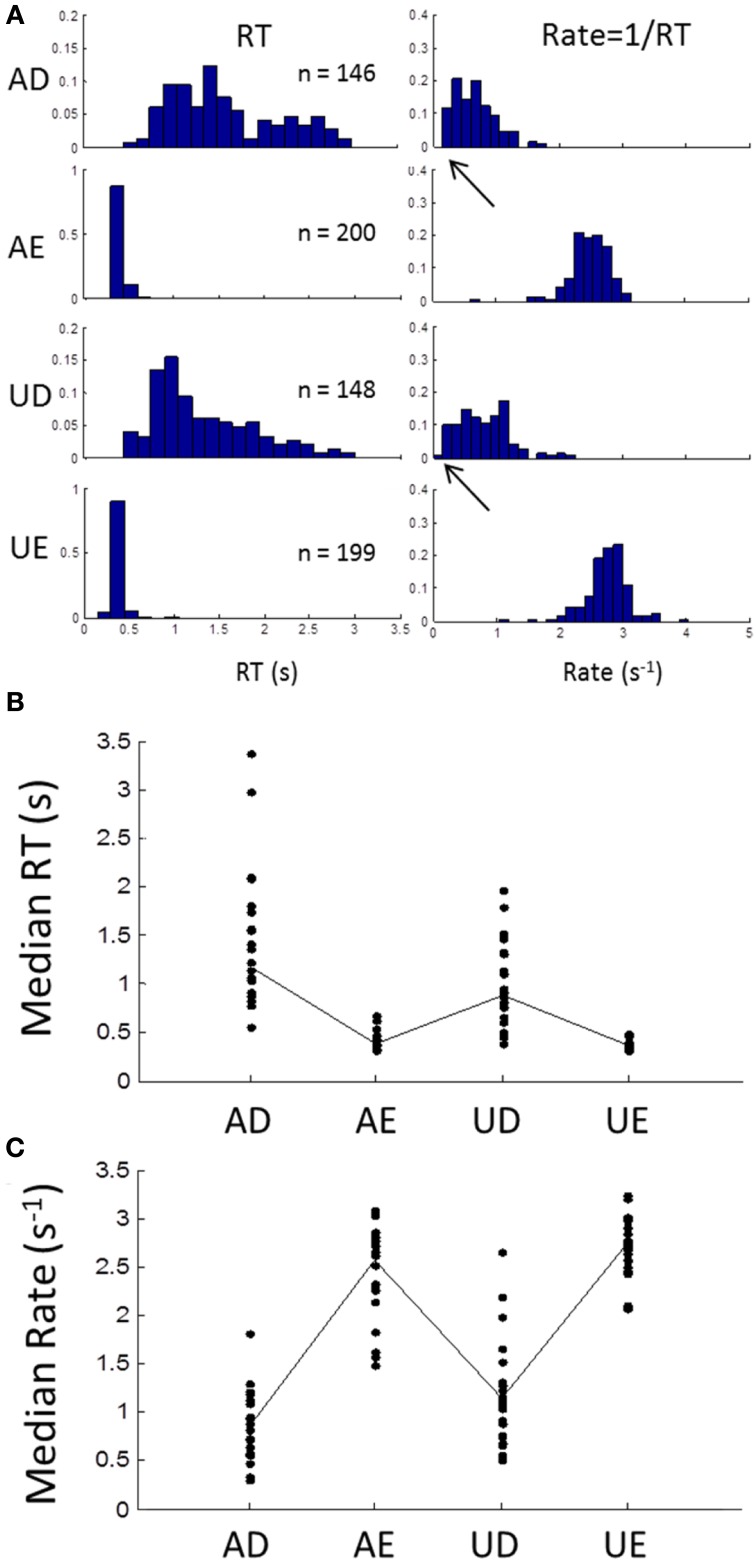
**(A)** An example of an individual subject's frequency distributions of RT (left column) and rate (right column) for the 4 different blocks (AD, accurate and difficult; AE, accurate and easy; UD, urgent and difficult; UE, urgent and easy; see Methods). In the easy tasks, RTs are brief with few errors (block size was 200 trials). For the difficult tasks RTs are much more variable with about 25% error rate. In the rate-domain, dispersion is similar for all blocks with a shift to lower rates for the difficult tasks. Note that the shift approaches zero (arrows) suggesting possible truncation. **(B)** Median RTs for all subjects showing longer RTs for difficult blocks and more inter-subject variability. **(C)** Same as **(B)** but for median rates showing similar inter-subject variability for all blocks.

Subjects' RTs were clearly sensitive to the task and stimulus manipulations, as shown by the example in Figure 2A (left column). When stimulus discriminability was easy, RT distributions were brief with low dispersion (AE and UE), but when difficult, they became longer and much more dispersive (AD and UD). In the rate-domain (reciprocal RT) difficulty resulted in a shift toward zero, but the dispersion remained similar (Figure 2A right column). For the difficult tasks, the rate distributions appear to approach zero and possibly became truncated. The difficulty was also evident by the number of errors (~25% in this example).

Similar patterns were seen in all subjects, as can be seen from the plot of medians of RT for all subjects in Figure 2B. Again there was much more inter-subject variability for the difficult tasks, but in the rate-domain the variability was more even (Figure 2C). Non-parametric testing (Wilcoxon test) showed that the medians differed significantly between the difficult and easy discriminability (AD∪UD vs. AE∪UE: *p* < 0.001), and between task instructions (AD∪AE vs. UD∪UE: *p* < 0.001).

**Figure 3:**
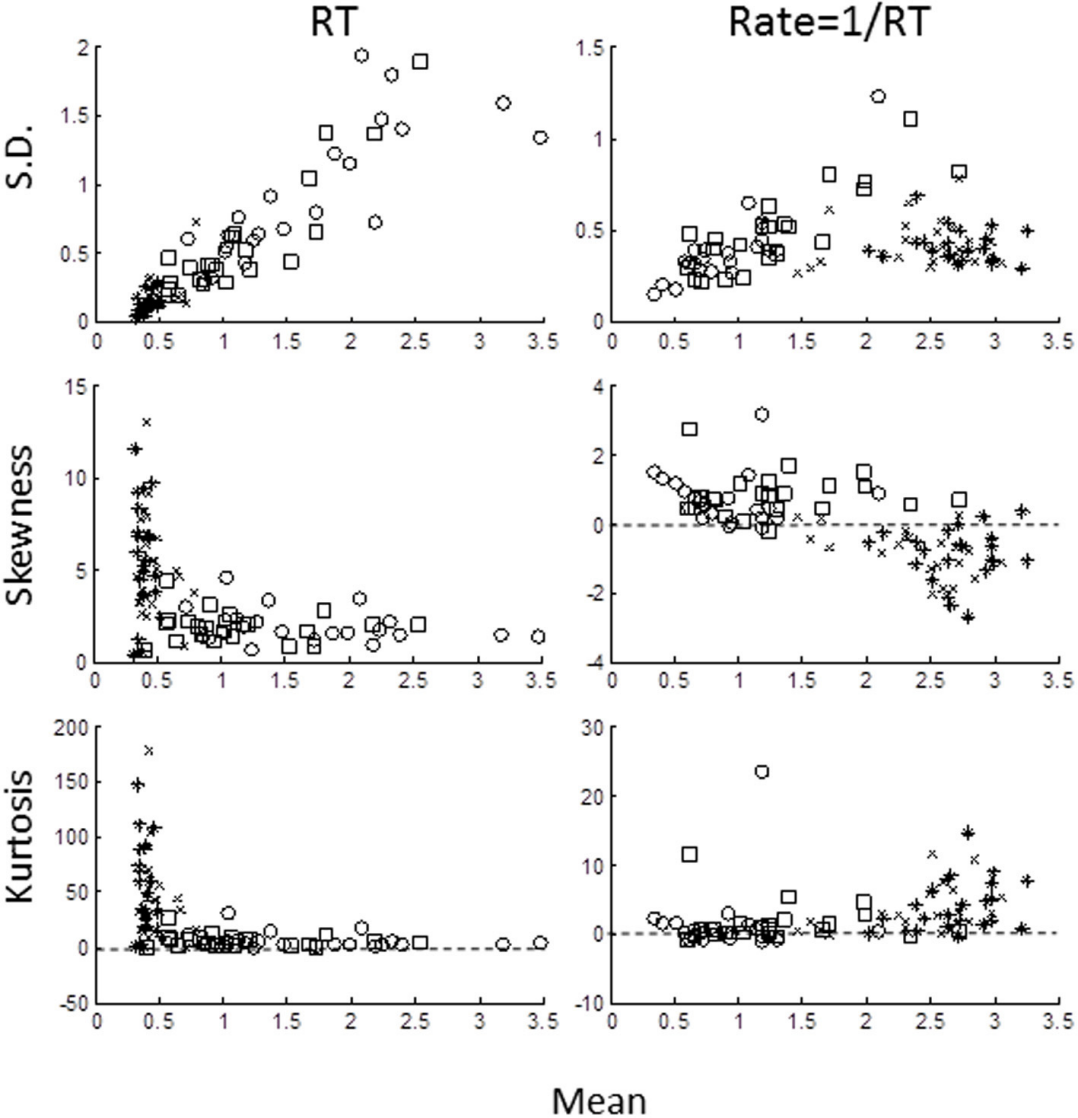
**Sample moments for RTs (left column) and rate (right column) plotted against mean for standard deviation (top row), skewness (middle row), and excess kurtosis (bottom row)**. Each symbol represents the moment for each block for each subject: AD- open circles; AE- crosses; UD- open squares; UE- asterisks). Note different in ordinate scales for RTs and rate moments.

We computed the sample central moments (mean, standard deviation, skewness, excess kurtosis) in the time- and rate-domains (Figure 3) for each task for each subject. In the time-domain (left column), the moments were strongly interdependent, as expected from skewed distributions. Standard deviation increased and skewness and excess kurtosis decreased with the mean (note that skewness and kurtosis are normalized with respect to standard deviation). In the rate-domain (right column), however, the interdependence was much weaker (note the difference in ordinate scales).

**Figure 4:**
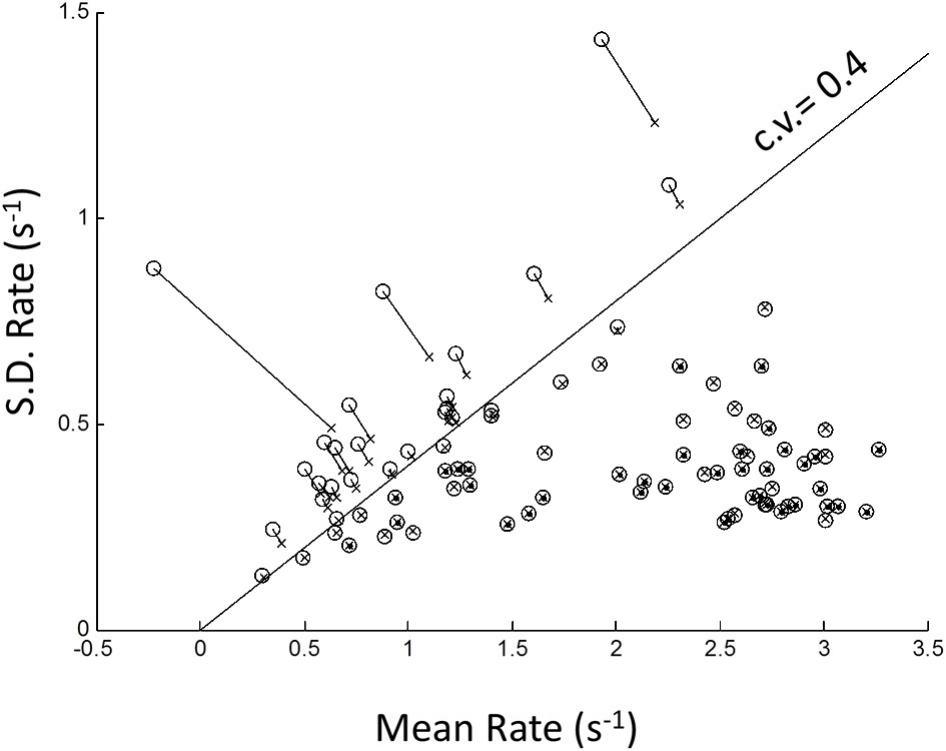
**Plot of standard deviation vs. mean of all blocks in the rate-domain**. Crosses indicate conventional sample moments (same as top-right panel in Figure 3). Circles indicate maximum likelihood estimates (MLE) of same blocks assuming a left truncated Normal. Line is *SD* = Mean/2.5. To the right of line sample moments coincide with rectrN MLE moments; to the left MLE moments shift to higher standard deviations and lower means (connecting lines).

Because of possible left truncation, we estimated the mean and standard deviation of the putative underlying Normal rate distribution using MLE (see Methods). We set the left truncation to 0.0167 s^−1^ corresponding to a time-out of 60 s (Figure 4). When the sample coefficient of variation (CV) was less than 0.4 (z-score = 2.5; line in Figure 4) the MLE estimates (circles) were seen to agree closely with sample moments (crosses). For higher CVs the MLE moments estimates were shifted from the conventional estimates (shown by up-left lines). These shifts in MLE moments are expected from left truncation, and are consistent with, but not definitive of an underlying truncated Normal distribution. Therefore, we next grouped blocks according whether their truncation was severe, “truncated” blocks (CV > 0.4), or negligible, “untruncated” blocks (CV < 0.4).

### Group distribution

**Figure 5:**
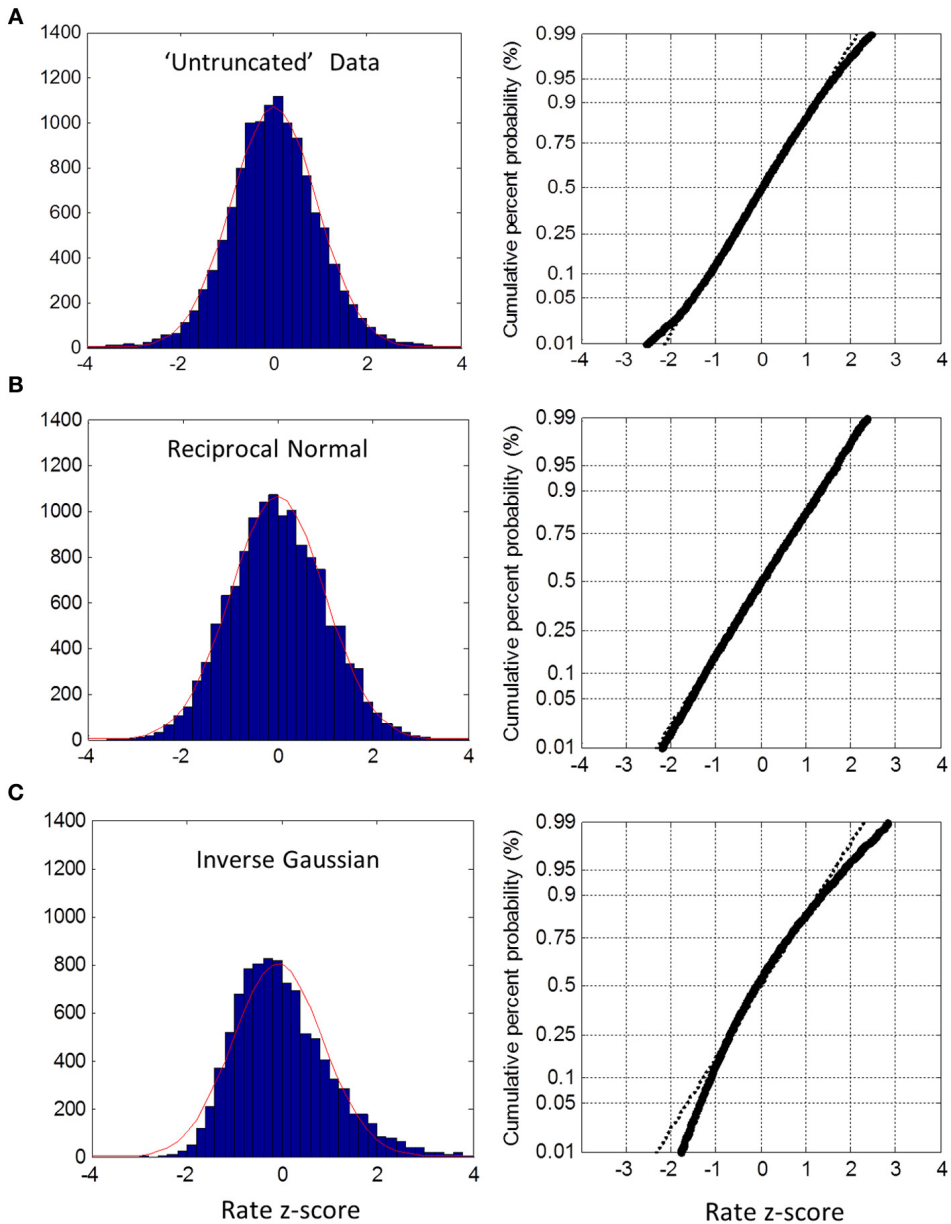
**Untruncated group rate histograms (left column) and rate probit plots (right column). (A)** Empirical rate from “untruncated” blocks (c.v. < 0.4) showing near Normal distribution over 5–95% interval with slight deviation in the tails **(B)** Simulated data using reciprocal Normal for RT distribution (see text) showing almost perfect Normal rate distribution. **(C)** Simulated data using inverse Gaussian for RT distributions showing obvious deviations from Normal rate.

In the untruncated blocks, we standardized the rate for each trial into a z-score based on the ML mean and standard deviation of its block, and then collapsed all trials into one group. The distribution of the untruncated group was very close to Normal between the 5th and 95th percentile, as seen from the probit plot (Figure 5A). There was a slight deviation in the tails. As a check on this method, we created simulated data sets using the true reciprocal Normal distribution with the same ML moments and sample sizes as the empirical data. Carrying out exactly the same analysis, the rate distribution was a perfect Normal—as expected (Figure 5B). As a further check, we also simulated the inverse Gaussian. Here there is no truncation issue, so we used sample moments and sample sizes to generate the simulated data. As seen in Figure 5C, the reciprocal distribution of the inverse Gaussian is skewed and does not fit the Normal—as expected (Harris and Waddington, 2012). Thus, we are confident that near Normality is not an artifact, but reflects the underlying distribution of the empirical rate distributions.

**Figure 6:**
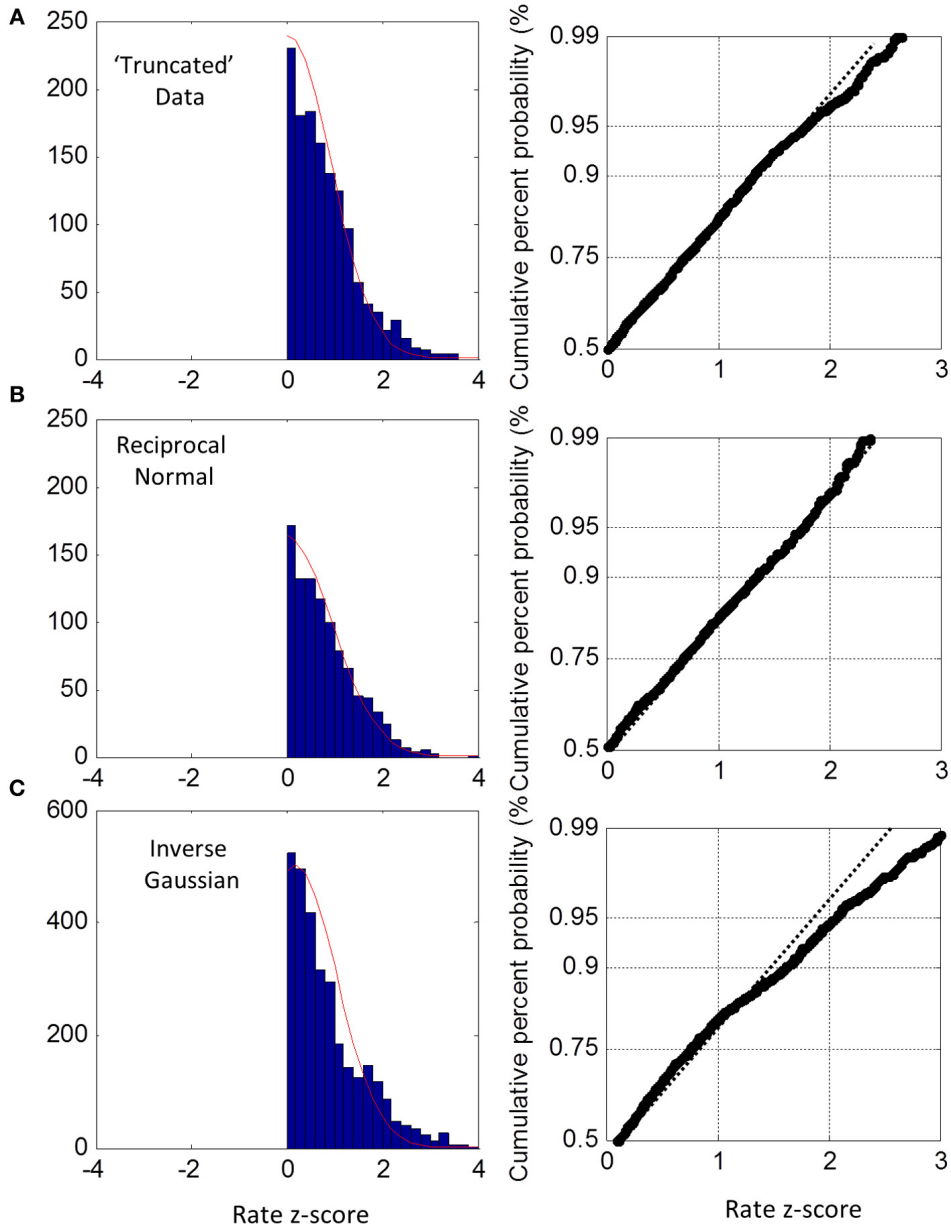
**Truncated group rate histograms (left column) and rate probit plots (right column), shown only for positive z-scores (see text). (A)** Empirical rate from “truncated” blocks (c.v. > 0.4) showing near Normal distribution over 50–95% percentiles; **(B)** simulated data using reciprocal Normal RTs showing near perfect Normal distributions (as expected). **(C)** Simulated data using inverse Gaussian RTs showing obvious deviations from Normal rate.

For the truncated blocks, we standardized as above using the ML mean and standard deviation and collapsed into one group. However, we only considered positive z-scores because any putative truncation would lead to under representation for negative z-scores (we included the one block that had a slightly negative ML mean, see Figure 3, but had no discernable effect on the plots when excluded). As shown in Figure 6A, the collapsed distribution was close to Normal with a slight deviation above the 95th percentile. Simulation with a true reciprocal Normal showed half a Normal distribution, as expected (Figure 6B), and the inverse Gaussian was not close to the truncated Normal (Figure 6C). Thus, we conclude that at least the right half of the truncated group are close to Normal, but not the inverse Gaussian. However, this does not address necessarily what happens near zero rate for each block (infra vide).

### Sequential dependency

#### Temporal effects

**Figure 7:**
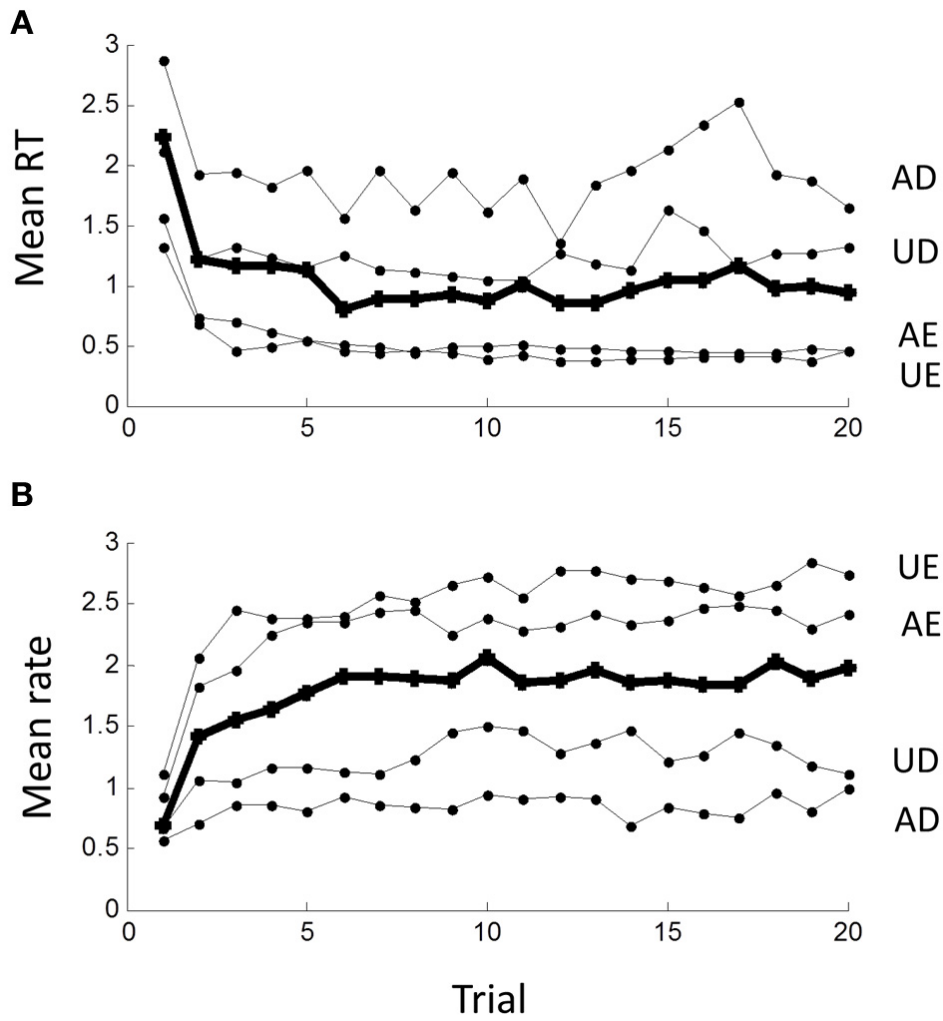
**Non-stationarity of responses in (A) the time-domain and (B) the rate-domain**. Means are computed across all subjects for the first 20 responses in each block; grand mean across conditions shown by thick line. Note initial transient lasting less than 10 trials, which is more pronounced for the AE and UE blocks.

The sequence of RTs during a block was clearly not statistically stationary as RTs were typically longer in the first few trials than later. This transient lasted less than 10 trials, after which a steady-state seemed to prevail, best seen by averaging across blocks in the time- or rate-domain (Figure 7). The transient was clearly more pronounced for the easy than difficult tasks.

We excluded the first 10 trials of each block in order to examine the steady-state component. The Pearson correlation coefficient between consecutive RTs was 0.20 with 63% of these being significant at *p* < 0.05. In the rate-domain this increased to 0.25 with 76% being significant.

**Figure 8:**
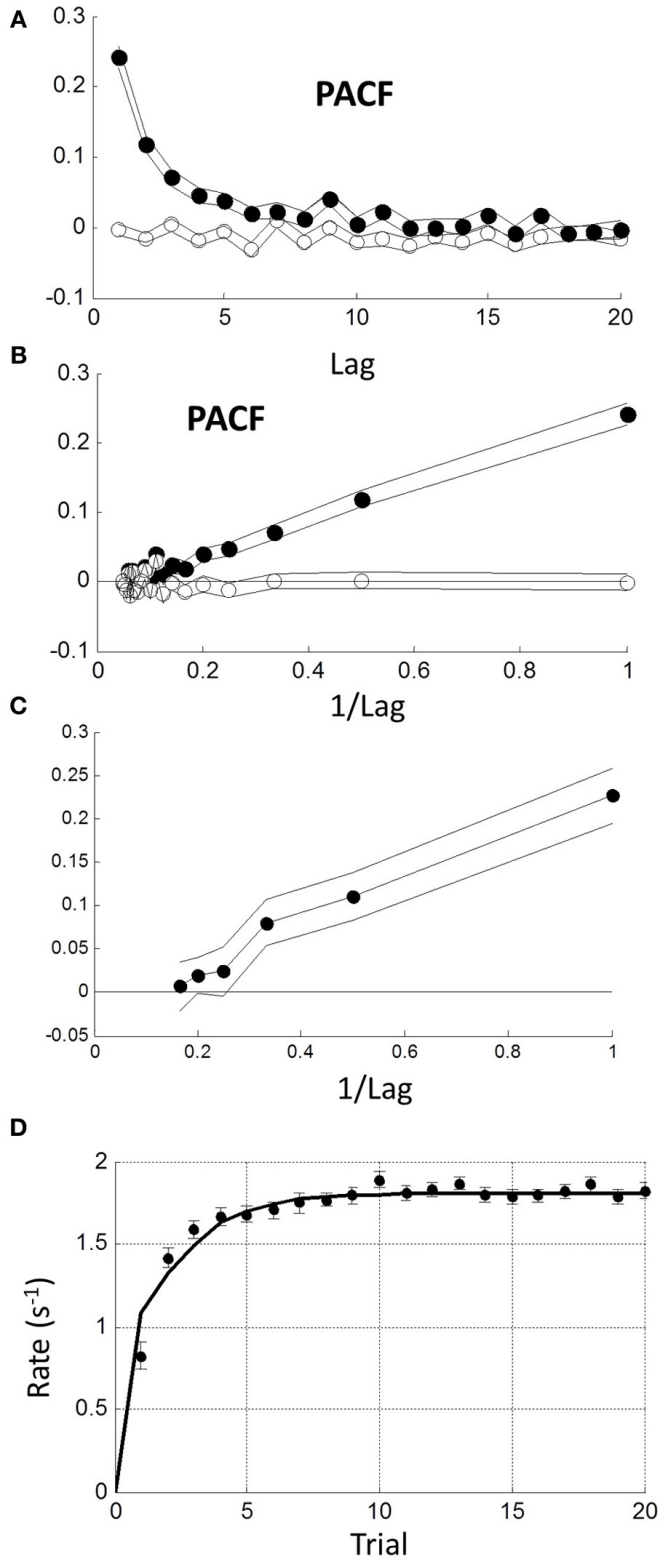
**Sequential dependency based on blocks without transients (first 10 trials omitted)**. **(A)** Mean partial autocorrelation function (PACF) of all blocks (filled symbols) showing smooth decay. Lines are ± 1 standard error. Open symbols show PACF for the same data after random shuffling leaving no sequential dependency. **(B)** PACF is plotted against reciprocal of lag showing a roughly linear increase (filled symbols). After de-correlation (see text) PACF coefficients become negligible (open symbols). **(C)** Maximum likelihood estimation of autoregressive coefficients (Equation 1.1) using the Box-Jenkins methods (see Methods) showing linear increase with reciprocal lag. **(D)** Comparison of step response function of autorgressive model (solid curve) with observed initial transient from grand mean in Figure 7B.

A 1-lag correlation would be expected to lead to autocorrelations with a geometric fall-off at higher lags. Therefore, we examined the partial autocorrelation function (PACF) to explore explicit dependencies up to lags of 20 (see Methods). The PACF of rate was positive and a smoothly decreasing function of lag with no obvious cut-off (Figure 8A filled circles). As a check, we shuffled trials randomly within each block and found no significant dependencies (Figure 8A open circles). When plotted against reciprocal lag, the PACF coefficients plot was approximately linear (Figure 8B; solid circles).

We next considered a stationary autoregressive (AR) relationship of the form: *r*_*n*_ = *a*_1_ *r*_*n* − 1_ + *a*_2_ *r*_*n* − 2_ + … + *a*_*m*_ *r*_*n* − *m*_ + *u*_*n*_ (see Equation 1.1 in Methods), where *a*_*i*_ (1 ≤ *i* ≤ *m*) are constant coefficients, *u*_*n*_ is a stochastic input on trial *n*, which we assumed stationary and Normal, and *m* is the order of the process (see Methods). We used the Box-Jenkins maximum likelihood estimation procedure (see Methods) to estimate the *a*_*i*_ for the first 6 lags. We only included “untruncated” blocks (CV < 0.4). Combining all such blocks revealed that only the first 3 weights were significantly different from zero and decreased roughly linearly with reciprocal lag *a*_1, 2, 3_ = {0.222, 0.104, 0.076}. The 4th weight *a*_4_ = 0.016 was borderline (Figure 8C). We also examined the difficult and easy tasks separately, but found negligible difference [AD∪UD: *a*_1, 2, 3, 4_ = {0.212, 0.100, 0.078, 0.016}; AE∪UE: *a*_1, 2, 3, 4_ = {0.227, 0.105, 0.076, 0.037}]. Henceforth, we used the first 3 weights of the combined tasks.

It is possible to invert the AR process to find the input, since from (1.1) we have *u*_*n*_ = *r*_*n*_ − *a*_1_ *r*_*n* − 1_ + *a*_2_ *r*_*n* − 2_ + … + *a*_*m*_ *r*_*n* − *m*_, and the resulting *u*_*n*_ should have no sequential dependency. To test this, we estimated the *u*_*n*_ sequence from each block and re-computed the mean PACF (Figure 8B open symbols). Clearly, sequential dependency was eliminated *on average* with a mean lag 1 correlation of 0.032. However, the number of blocks that had a significant lag 1 correlation also dropped from 61 to 10%—which is close to that expected by chance. This implies that most blocks were driven by a similar AR process.

The AR model in (1.1) has a step response which reflects the underlying dynamics behind the steady-state response. It is easily computed (curve in Figure 8D) and clearly similar to the empirical average transient response at the beginning of each block (grand average from Figure 7B). Thus, the transient response is consistent with the steady-state dynamics.

**Figure 9:**
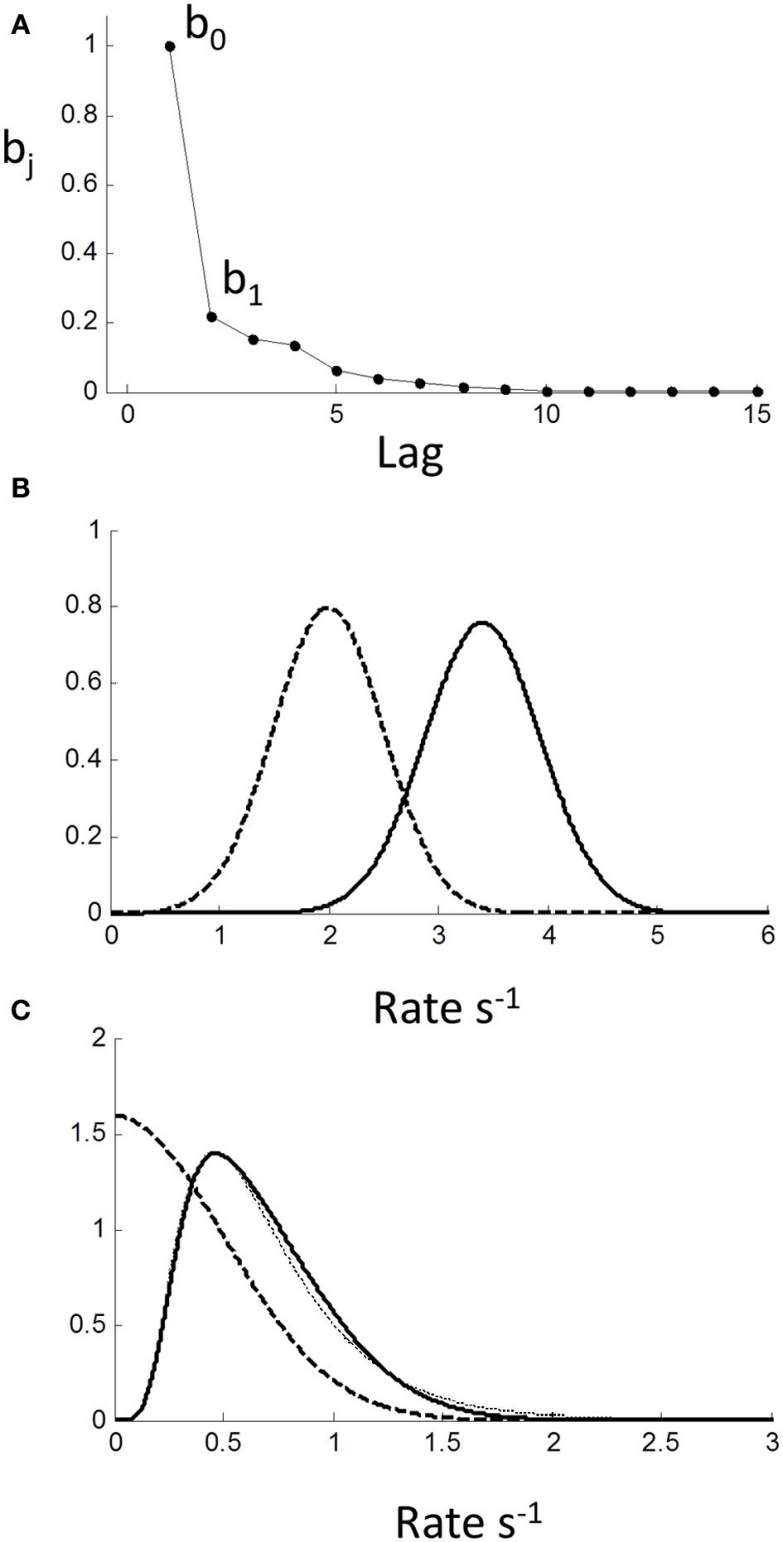
**Moving average (MA) model (Equation 1.2) computed from autoregressive coefficients using z-transform method (see Methods). (A)** MA coefficients show extended dependency on lag indicating memory of input. **(B)** Effect of MA on a Normal input distribution with minimal truncation. Input (μ = 2,σ = 0.3) (dashed curve) is shifted to higher rate (solid curve) with little change in shape; **(C)** Input is Normal (μ = 0,σ = 0.3) truncated at 0. Note truncation is eliminated by smoothing. Resulting pdf could be mistaken for a reciprocal inverse Gaussian distribution (dotted curve).

Using the single-sided z-transform, we converted (1.1) to a moving average (MA) formulation in terms of a discrete series of independent stochastic inputs *u*_*j*_ 1 ≤ *j* ≤ *n* (see Equation 1.2 in Methods): *r*_*n*_ = *b*_0_ *u*_*n*_ + *b*_1_ *u*_*n* − 1_ + *b*_2_ *u*_*n* − 2_ + …. The weights are the feed-forward impulse response function and are plotted against lag in Figure 9A. As can be seen, there is modest but prolonged dependence on input value history implying considerable “memory.”

Assuming stationarity, one effect of the sequential dependency is to scale the moments of the input (see Methods). Based on the AR weights, the mean of rate was *r* = 1.67*u*. The effect on standard deviation was small σ _*r*_ = 1.05(σ _*u*_), and on higher moments it was negligible. For an untruncated rate distribution, the effect of sequential dependency was to shift the rate distribution to the right with minimal changes to the shape of the distribution. Thus, we conclude the observed near-Normality of untruncated rate distributions is not a manifestation of the central limit theorem arising from the sequential dependency, but must reflect the near-Normality of the input distribution itself. Therefore, assuming the pdf of the input *p*_*u*_ (*r*) to be Normal, the output pdf *p*_*r*_ (*r*) can be computed numerically from the convolution sequence in (Equation 1.3) (see Methods). For an “untruncated” Normal input there is a shift to higher rate with negligible change in variance, as illustrated in Figure 9B. For an input truncated at zero, there is not only a shift in the mean, but the sharp truncation at zero is smoothed and eliminated (which can also be demonstrated analytically; see Methods). Remarkably, this smooth shape can also be fit very well by a reciprocal inverse Gaussian (dotted curve) when the tail is excluded (see Discussion).

#### Spatial effects

Previous studies have shown that mean RT can depend on the sequence of the laterality of previous trials (see Introduction), in particular whether laterality was repeated (**R**) or alternated (**A**). Thus, the sequence **RRRR** indicates that the stimulus and the previous four stimuli were all on the same side (i.e., all left *LLLLL* or all right *RRRRR*), whereas the sequence **AAAA** means that each stimulus alternated sides from the previous (*RLRLR* or *LRLRL*) (note the last symbol is the current trial). Jentzsch and Sommer (2002) examined sequences with 4 lags and showed a significant dependence of RT on a binary weighting of the AR sequence, where **R** was binary “0” and **A** binary “1” (e.g., **RRRR** = 0, **RRAR** = 2, **AARA** = 13, **AAAA** = 16). We used the same scheme for comparison.

For the easy tasks (AE and UE), averaging across all blocks showed a significant dependence on the AR sequence [*F*_(15, 645)_ = 4.58; *p* < 0.001] when all trials in a block were considered. In particular the sequences AARR, RRRA, RRRA, were associated with high RTs (arrow in Figure 8), and remarkably similar to Jentzsch and Sommer's results. The inverse pattern was more clearly seen in the rate-domain, with smaller and more even standard errors. For the difficult tasks (AD and UD), there was no significant pattern in the time- or rate-domain.

## Discussion

These data clearly show that when the task is easy (AE and UE blocks), RT distributions are close to reciprocal Normal, and not close to the inverse Gaussian distribution. Moreover, we have demonstrated this using practical block sizes (*n* = 200) collapsed across 24 subjects after standardization, unlike previous studies which used very large data sets recorded from only a few subjects. We emphasize that this near-Normality of rate was not an artifact from collapsing across subjects, as this does not invoke the central limit theorem, but simply combines the underlying distributions—as confirmed by Monte-Carlo simulations (Figure 5B). We conclude that 2-alternative choice manual RT distributions are very close to the rectrN distribution, similar to the simple reaction experiments with saccades (Carpenter, 1981; Carpenter and Williams, 1995; Reddi and Carpenter, 2000) and the few studies of *simple* manual reaction times (Carpenter, 1999; Harris and Waddington, 2012). In simple RT studies it is necessary to introduce a variable foreperiod to prevent anticipation for the stimulus onset. In choice RT study, a foreperiod may increase “preparedness,” but randomization is not essential, as a choice cannot be made with confidence until the discriminative stimulus appears, and Bertelson and Tisseyre (1968) have shown similar effects for constant or random foreperiods in choice experiments. We chose a constant foreperiod to reduce the amount of extrinsic variability introduced into the decision process (see Methods). We can conclude that near-Normality in the rate domain is not a consequence of foreperiod randomization, and by implication presumably neither in simple RT experiments. However, this does not eliminate a possible role of a subject's intrinsic variability in judging foreperiod durations (i.e., Weber's law), and whether or how this affects the rate distribution remains to be explored.

It is difficult to reconcile the rectrN with a pure Wiener diffusion process, where within trial drift noise is Normal (Figure 1B), as this would yield an inverse Gaussian distribution in the time-domain, or a reciprocal inverse Gaussian in the rate-domain. Monte Carlo simulation using the reciprocal inverse Gaussian with moments from our subjects did not yield near Normal rates (Figure 5C). Ratcliff (1978) considered the compound inverse Gaussian where drift rate fluctuated between trials with another Normal distribution. This would fit the reciprocal Normal if there were no drift noise, which is consistent with Carpenter's LATER model. This strongly suggests that the underlying RT process operates in the rate-domain, rather than in the more intuitive time-domain. It also explains why RTs are so variable—modest symmetric fluctuations in rate can lead to asymmetric and very high changes in RT, especially when rate becomes small as occurs in difficult tasks.

Temporal sequential dependency among trials has frequently been observed in choice reaction experiments (Laming, 1979). Clearly, any inter-trial correlations affect between-trial fluctuations, but they have been ignored in recent models of RT distributions. Using autoregressive techniques, we have shown explicit dependency of rate output for at least the 3 previous trials, very similar to Laming's original finding in the time-domain. Converting to a MA representation, this “memory” extends even further in terms of stimulus inputs (Figure 9A). We also found a transient response at the beginning of each block lasting less than 10 trials, which was similar to the predicted step response of the steady-state dynamics (Figure 8D). The simplest explanation is that the rest time between blocks allowed the memory “trace” to decay. However, this needs further exploration since we did not manipulate block intervals, and it was not possible to distinguish between sequential dependencies that are based on absolute time or based on trial number.

Based on moments, the main effect of this temporal dependency was to scale the mean response rate to higher values (i.e., shorten RTs) with little change in variance or higher moments (Figure 9B). One could view this as improving signal-noise ratio, or that previous trials/stimuli provide some information about the upcoming stimulus (prediction), hence allowing a faster response. Because higher moments are negligibly affected by the MA process, we can also conclude that the temporal sequential dependency does not cause rate to be Normal via the central limit theorem, and we deduce that the input must already be near-Normal.

**Figure 10:**
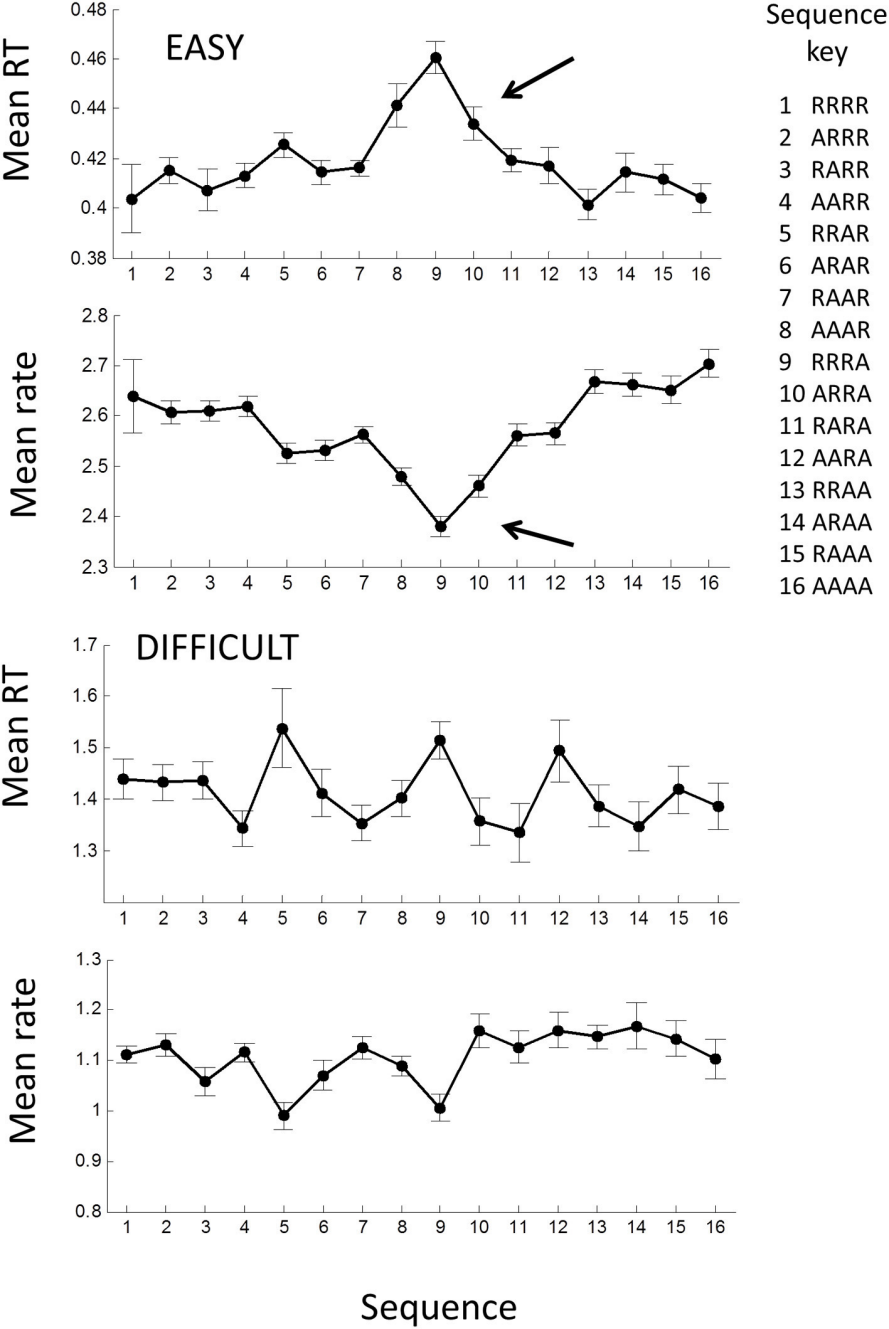
**Mean response time and rate plotted against the laterality sequence of previous 4 trials: R, laterality repeated; A, laterality alternated (after Jentzsch and Sommer, 2002)**. Means were computed across all AE and UE blocks; error bars are ± 1 *within* standard error. Filled symbols show means for all trials in each block; note the significant increase in RT for RRRA sequence (row). A similar picture is seen in the rate-domain.

We also found a sequential dependency that was related to the sequence of stimulus laterality for the easy tasks. Using Jentsch and Sommer's binary weighting system, we found a remarkably similar result to theirs for the easy tasks with **RRRR** and **AAAA** having the highest rates (shortest RTs) and **AAAR**, **RRRA**, **ARRA** having the lowest rates (longest RTs) (Figure 10). The weighting scheme of Jentsch and Sommer's extends backward for 4 lags and assumes binary (power function) weighting. From the temporal viewpoint, our results suggest that the 4th lag is questionable and that weightings should follow an approximately hyperbolic decrease. Using this scheme, the dependency becomes even more pronounced (not shown). It is tempting to argue that the temporal and spatial dependencies are manifestations of the same process. Jentsch and Sommer have assumed the dependency reflects a decaying memory trace, as this would explain why higher-order dependencies tend to be weaker when the trials are longer in absolute time. Indeed, we found that the spatial dependency was absent in the difficult tasks (Figure 10). Surprisingly, the temporal dependency was still present and virtually identical to the easy task AR process. The reason for this is unclear at present, but suggests that temporal and spatial dependencies can be dissociated.

We emphasize that we have examined sequential dependency in the rate-domain. In the rate domain, a sequence of responses is a well-behaved stochastic process because of its near-Normality, and this permits the wide range of standard analysis techniques (moments, autocorrelations, spectral analyses, etc). In the time-domain this is not necessarily the case because taking the reciprocal of rate is a non-linear operation. Trials with low rates become disproportionately magnified in the time domain, which can lead to “spikes” with very long RTs. In particular, there is the possibility that artefacts may arise in power spectra as these spikes have high spectral energy, and we advocate caution interpreting power spectra based only on time-domain analyses (e.g., 1/f noise: Thornton and Gilden, 2005) subject to further exploration.

### Truncation

Strictly, the Normal distribution has infinite extent and includes zero and negative rates, but this is not possible in RT experiments, so we need to consider the left-truncated Normal and the corresponding reciprocal truncated Normal (Harris and Waddington, 2012). We observed that when the task became more difficult (AD and UD), there was a leftward shift of the rate distribution (i.e., longer RTs) (Figure 2A) suggesting that left-truncation may have occurred. Because moments are sensitive to truncation, we used MLE to find the underlying Normal that fitted each block the best, and this showed that truncation was occurring (Figure 4). Collapsing across these subjects showed that the untruncated right half of the distribution was also very close to Normal (Figure 6A). This is a novel finding, and is evidence that task difficulty can lead to truncated Normal rate distributions. This has not been considered in previous models but has some far-reaching implications.

Truncation leads to very long RTs, which could theoretically approach infinity. Such responses would not usually be observed because either the experimenter imposes a maximum trial duration (time-out), or because the experiment is of finite duration in time or in number of trials. Thus, practically, rate will appear bound at some non-zero minimum, depending of the experimental design (see Harris and Waddington, 2012 for further discussion). For easy tasks, this will have minimal effect since long RTs are rare, but as the task becomes more difficult, the effect of truncation becomes increasingly important.

Interestingly, it has been proposed that the latency distribution of saccades departs from reciprocal normal for low stimulus contrasts, and that the inverse Gaussian is a better model (Carpenter et al., 2009). However, could this instead be due to truncation of the reciprocal Normal? Consider the theoretical example in Figure 9C, where we have set the rate standard deviation to 0.3 s^−1^ with left truncation set by a mean of 0. The effect of temporal sequential dependency is to smooth out the truncation, which reduces the probability of very long RTs. The resulting pdf could easily be mistaken for the reciprocal inverse Gaussian (Figure 9C dotted curve). Thus, in the time-domain, it is plausible that studies using the inverse Gaussian may have overlooked the reciprocal truncated Normal with sequential dependency as a more parsimonious and unifying explanation.

### Non-homogeneity

In this experiment we have used homogenous and stationary blocks, where the same stimuli were used in each trial of a block, and the laterality was random. However, many RT experiments are not homogenous, and the stimulus value changes on trials within a block. Generally, we expect that rate would no longer be reciprocal Normal. We distinguish between discrete and continuous non-homogeneity.

In the discrete case, a block contains a small number of different but known stimuli that are typically randomized or counterbalanced within the block. Assuming independent trials, the observed rate on each trial would then be a single sample from the Normal distribution associated with that stimulus. The overall rate distribution would then be a mixture of Normal distributions depending on the value and relative frequency of each stimulus. Since the stimulus is known on each trial, responses could be segregated and the rate distributions computed. Clearly, any sequential dependency should be reduced before segregation.

The continuous case is more problematic. It typically occurs when task difficulty and/or stimulus value vary on every trial in an unknown way. The rate on each trial can still be considered as a single sample from a Normal distribution, but the mean of the rate distribution (and possibly the standard deviation) are continuously variable leading overall to a compound Normal distribution, which can take on a wide range of positively or negatively skewed shapes. Whether de-convolving a putative Normal distribution is useful remains to be explored on real data.

### Rate and optimality

As posed in the introduction, why RTs are so variable and whether, or under what circumstances, they could be optimal are longstanding questions that have been asked or assumed to be answerable by time-domain analysis (e.g., Luce, 1986; Bogacz et al., 2006). However, our and Carpenter's data are highly suggestive that there exists a preferred rate, r^*^, for a given set of experimental conditions, and that rate fluctuates according to a Normal random process from trial to trial around r^*^. Clearly, modest symmetrical variations in rate can lead to very large and highly asymmetric fluctuations in the time domain, especially when r^*^ is small—as occurs in difficult discriminative tasks. Also r^*^ is easily recognizable as the modal rate, but there is no obvious landmark in the time domain: t^*^ = 1/r^*^ does not correspond to the mode in the time-domain. Moreover, the rectrN is a strange distribution without finite moments (Harris and Waddington, 2012), whereas the Normal distribution is a common basic distribution. This strongly suggests that we should be considering rate as the more fundamental variable than RT, even if it seems counter-intuitive.

It seems that if we accept a rise to threshold model, then we require a deterministic drift rate that fluctuates *between* trials with a truncated Normal distribution, as originally proposed by Carpenter (1981). It is conceivable that there is still a stochastic rise to threshold, but it would need to be almost completely masked by the inter-trial variability (this needs future modeling), and rate is still the dominant variable. However, it is important not to conflate proximal with ultimate explanations. At the proximal level, there must be some physiological mechanism for triggering an all-or-none response, and an accumulator process seems physiologically plausible. However, even if true, it only explains how rate could be represented mechanistically, and there is a myriad of ways in which an accumulator could be constructed/evolved as a trigger (e.g., linear vs. curvilinear signal rise, deterministic vs. stochastic signal, fixed vs. variable trigger level; Figure 1A). It does not explain why rate is important.

Rate of response may be fundamental for an organism. For example, in the study of natural foraging, it is widely assumed that animals seek to maximize the rate of nutrient intake, rather than quantity *per se*. This has led to the marginal value theorem (Charnov, 1976) which predicts the time spent by animals on patches of food. In the study of animal learning, Skinner introduced his famous cumulative plots as a way of visualizing the stationarity of an animal's rate of response (Skinner, 1938; Ferster and Skinner, 1957). There is an obvious parallel between RT and operant behavior. When a subject presses a button (“operant”), she presumably derives a reward if the button press is a “correct” response, and a loss if “incorrect.” The onset of lights acts as a “discriminant” or “conditioned” stimulus that provides information about the probability of reward (Skinner, 1938). It is well known that response times decrease with increasing reward but also increasing intensity of the conditioned stimulus (Mackintosh, 1974). Similarly, numerous studies have shown RTs decrease with increasing reward (Takikawa et al., 2002; Lauwereyns and Wisnewski, 2006; Spreckelmeyer et al., 2009; Milstein and Dorris, 2011; Delmonte et al., 2012; van Hell et al., 2012; Gopin et al., 2013) or increasing stimulus intensity (Cattell, 1886; Piéron, 1914). This leads us to consider the possibility of maximizing expected *rate* of reward or utility as an explanation for our observations (also considered by Gold and Shadlen, 2002).

**Figure 11:**
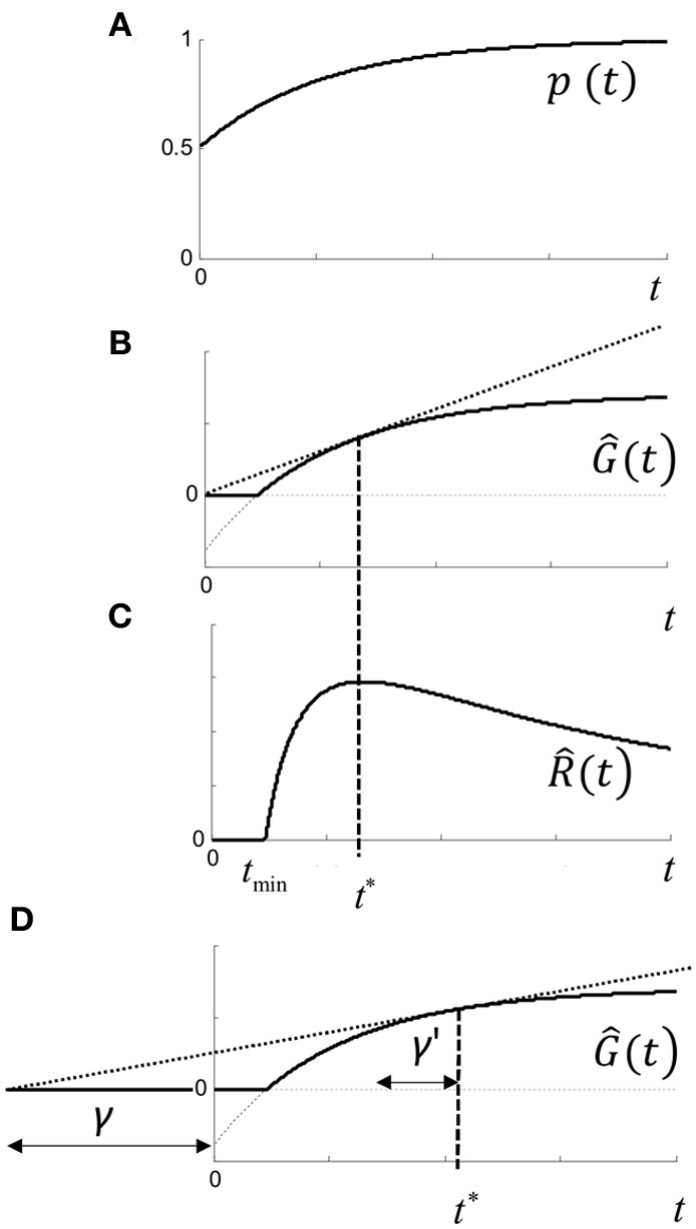
**Rate model. (A)** *p*(*t*) is subjective probability of being correct given a response is made at time *t*, and is assumed to be concave. Initial value of *p*(*t*) assumes guessing with no prior information, and final value is assumes that response will be correct given infinite time. **(B)** *Ĝ*(*t*) is the expected gain in utility (Equation 1.4) for a response made at time *t*. Note that gain may be negative (i.e., loss) for *t* < *t*_min_ (dashed curve) and no response is optimal. **(C)** $\hat{R}$(*t*) is the expected rate of gain in utility (Equation 1.5) which has a maximum at t^*^, and can be visualized geometrically as the point where the tangent touches *Ĝ*(*t*) in **(B**,**D)** shifting the time origin back by γ increases t^*^ by γ′ (see text).

For each trial, we define the gain in subjective utility for a correct response by *U*^+^ > 0, and the loss by *U*^−^ > 0. Objectively, utility would be maximized by responding to the correct stimulus any time after the stimulus onset. The stimulus value depends on the temporal response of the visual system, and will also increase in time due to any temporal integration and/or Bayesian update of priors. We therefore denote *p*(*t*) as the subjective probability of making a correct response given that a response occurs at *t* (measured relative to some origin; see below). We assume that *p*(*t*) is a concave function (Figure 11A), where for two alternatives with no prior information, *p*(0) = 0.5.

The expected gain in utility *Ĝ*(*t*) for a response at time *t* is (curve in Figure 11B):

(1.4) $$\hat{G}(t)=U^{+}p(t)-U^{-}\left(1-p(t)\right)=\left(U^{+}+U^{-}\right)p(t)-U^{-}$$

It can be seen that expected gain will be negative when *t* < *t*_min_, where *p*(*t*_min_) = 1/(*U*^+^ /*U*^−^ +1). In this case, it does not pay to respond at all, but there will always be a positive gain as *p*(*t*) → 1 and maximized by responding as late as possible. Expected rate of gain is $\hat{R}$(*t*) = *Ĝ*(*t*)/*t*. When rate of gain is positive, there may be an optimal time to respond given by t^*^ = $\hat{R}$(*t*), which is the solution to:

(1.5) $$\begin{array}{l} t^{*}=\frac{\hat{G}(t^{*})}{{\hat{G}}^{\prime }(t^{*})} \end{array}$$

where the dash refers to the derivative with respect to *t* (Figure 11C). The conditions for a positive maximum are complicated, but it occurs under quite broad conditions and is easily visualized geometrically in Figure 11B, since from (1.4) the optimum is given by the tangent of *Ĝ*(*t*) that intercepts the origin. Thus, depending on the utility payoff ratio *U*^+^ /*U*^−^, and *p*(*t*), there is an optimal time to respond. *Responding as quickly as possible is generally suboptimal*—it pays to wait for a specific time to respond.

We can make some general deductions. First, any increase/decrease in the utility payoff ratio, *U*^+^ /*U*^−^, will reduce/increase t^*^ for a concave *p*(*t*). Thus, increasing reward will reduce t^*^, as empirically observed (*vide supra*). In our experiment, asking subjects to respond accurately as opposed to quickly required “caution” by reducing the ratio and increasing t^*^ (Figure 2).

Faster/slower rise in *p*(*t*) will also reduce/increase t^*^ similar to, but not in precisely the same manner as manipulating payoff. For example, increasing the number of alternatives, *n*, will reduce *p*(*t*) since *p*(0) = 1/*n* (given no other prior information) and hence increase t^*^. Whether there is a logarithmic relationship between *n* and E[t^*^] (Hick's law) depends on the precise form of *p*(*t*) and remains to be explored. On the other hand, any prior information will decrease the rise-time of *p*(*t*) and reduce t^*^, as has been reported in some experiments with random foreperiods (see Niemi and Näätänen, 1981).

Stimulus intensity has a strong inverse relationship on t^*^, but this depends on *p*(*t*). The simplest way to parameterize *p*(*t*), is to assume that *p*(*t*) depends on a single parameter, ε, that accelerates time so that *p*_ε_ (*t*) = *p*(ε *t*). We assume that $\hat{\varepsilon }$ is an unbiased estimate of ε and distributed Normally across trials. It follows that *Ĝ*_$\hat{\varepsilon }$_ (*t*) = *Ĝ*($\hat{\varepsilon }$ *t*) and *Ĝ*'_$\hat{\varepsilon }$_ (*t*) = $\hat{\varepsilon }$ *Ĝ*($\hat{\varepsilon }$*t*). Then (1.5) becomes

(1.6) $${\hat{G}}^{\prime }(\hat{\varepsilon }t^{*})=\frac{\hat{G}(\hat{\varepsilon }t^{*})}{\hat{\varepsilon }t^{*}}$$

so it follows that the optimal solution t^*^ is given by:

(1.7) $$t^{*}=\frac{t_{1}}{\hat{\varepsilon }}$$

where *t*_1_ is the solution to (1.6) evaluated at $\hat{\varepsilon }$ = 1. Thus, if each trial is optimized based on the estimate $\hat{\varepsilon }$, then the optimal time to respond is distributed with the reciprocal of the distribution of $\hat{\varepsilon }$ and hence has a reciprocal Normal distribution, as observed.

Since only one reward can occur per trial, we would expect trial duration to be the more relevant epoch for response rate, rather than decision time *per se*. Including an additional non-decision time *T*_*ND*_ (foreperiod, sensorimotor delays, etc.) in the computation of estimated rate: $\hat{R}$(*t*) = *Ĝ*(*t*)/(*t* + *T*_*ND*_) yields the more general equation for t^*^

(1.8) $$t^{*}+T_{ND}=\frac{\hat{G}(t^{*})}{{\hat{G}}^{\prime }(t^{*})}$$

As shown in Figure 11D, including *T*_*ND*_ increases optimal response time (relative to stimulus onset). In other words, decision time depends on the amount of non-decision time. Returning to the parametric model: *Ĝ*_$\hat{\varepsilon }$_ (*t*) = *Ĝ*($\hat{\varepsilon }$ *t*), we note that

(1.9) $$\hat{\varepsilon }\left(t^{*}+T_{ND}\right)=\frac{\hat{G}(\hat{\varepsilon }t^{*})}{{\hat{G}}^{\prime }(\hat{\varepsilon }t^{*})}$$

**Figure 12:**
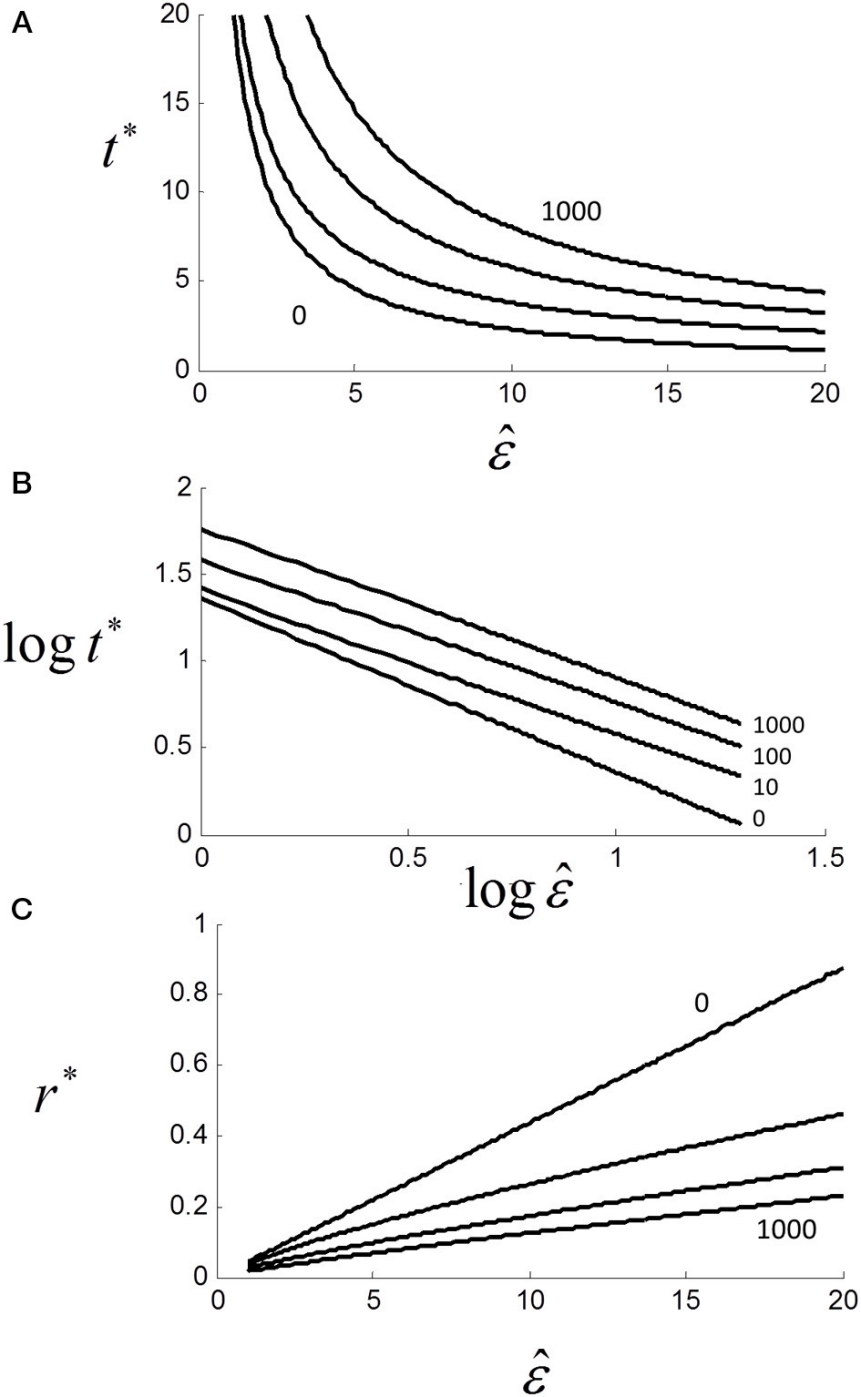
**(A)** Effect of scaling factor $\hat{\varepsilon }$ on optimal decision time t^*^ for different non-decision time *T*_*ND*_ = {0,10,100,1000} (see text). Note that t^*^ and hence RT increases with *T*_*ND*_, although asymptote is zero (not shown); **(B)** same as **(A)** but on log-log axes (base 10) showing near power function t^*^ ≈ aε ^−*k*^ with *k* = {0, 0.82, 0.83, 0.87} and *a* = {25.1, 25.1, 39.8, 63.1} from linear regressions; **(C)** linear plot of optimal rate r^*^ vs. $\hat{\varepsilon }$. Although strictly a power function, relationship is locally quasi-linear.

The solution is not the same as for (1.6), and requires an explicit form for *p*(*t*). For the purposes of illustration, we assumed a simple exponential form of *p*(*t*) = 1/2 + (1 − exp (− $\hat{\varepsilon }$*t*))/2 and plotted t^*^ against $\hat{\varepsilon }$ with *U*^+^ = 1, *U*^−^ = 5 and parametric in *T*_*ND*_ (Figure 12A). As can be seen, t^*^ decays with increasing $\hat{\varepsilon }$ but also increases with *T*_*ND*_. Although we did not manipulate “non-decision” time here, others have shown that increasing foreperiod increases RT in both simple (Niemi and Näätänen, 1981) and choice RT (Green et al., 1983).

For *T*_*ND*_ > 0, the relationship is still very close to a power law with t^*^ ≈ *a*ε ^−*k*^ where *k* ≈ 0.8 (Figure 12B). In terms of rate, we can see that as *T*_*ND*_ increases, r^*^ decreases but the relationship to $\hat{\varepsilon }$ is still locally close to linear even for very large *T*_*ND*_ (Figure 12C). Thus, if $\hat{\varepsilon }$ is Normally distributed r^*^ will also be very near Normal.

If we add sensorimotor delays γ to decision time, then we have *RT* = *a*$\hat{\varepsilon }$ ^−*k*^ + γ which is clearly similar to Pieron's law: *E*[*RT*] = α *I*^− β^ + γ, where α,β and γ are constants for a given experiment and *I* is objective stimulus intensity. Piéron's law was originally found for simple RT experiments, but also holds for choice RTs (van Maanen et al., 2012). If we assume that $\hat{\varepsilon }$ is subjective estimated stimulus intensity, then we require $\hat{\varepsilon }$ ∝ *I*^β /*k*^ which is plausible from Steven's power law (Chater and Brown, 1999).

### Mechanism

How optimal rate could be controlled is open to speculation. We can see that the mechanism in Figure 1A could act as an equation solver since the time of crossing is the solution of ρ (*t*) = θ (*t*) [more formally: the lowest real positive root of ρ (*t*) − θ (*t*)], and when equality is reached, the behavior is triggered in real-time. This can be mapped onto (1.5) in an infinity of ways. A simple possibility is that a deterministic linear rise to threshold behaves as rate-to-time converter (Figure 1C). The input $\hat{R}$(*t*) is integrated in time to yield a rising deterministic ρ (*t*) which triggers the response when then a threshold is reached. Gold and Shadlen (2002) proposed that an optimal decision time could be found by an adaptive process (trial-and-error) that varies the threshold. In this case, the distribution of decision times would be given by the distribution of thresholds (for a fixed ρ (*t*)), but this hardly explains why RTs have a near-rectrN distribution. A more parsimonious model would be that the optimal ρ (*t*) is found for a fixed threshold (i.e., Carpenter's original model). Normally distributed estimates of ρ (*t*) would then yield RTs with the observed rectrN distribution. It is possible that both threshold and ρ (*t*) are variable leading to a ratio of distributions for decision time (Waddington and Harris, 2013), although we have no evidence for this in this experiment.

Taking a different perspective, we can draw a correspondence between rate (responses per second) and frequency (cycles per second), and consider control by underlying banks of oscillators in the Fourier domain. It is conceivable that repetitive nature of RT experiments entrain oscillator frequencies, possibly with phase resets from the stimulus onset to allow some degree of prediction. Our observed temporal and spatial sequential dependencies could reflect this entrainment (phase-locking), and the Normal distribution of rate could reflect sampling of subpopulations of oscillators. This is speculative, but not discordant with the known correlation between RTs and alpha brain waves (Drewes and van Rullen, 2011; Diederich et al., 2012; Hamm et al., 2012).

## Summary

For 2-alternative manual choice RTs, distributions are close to the reciprocal Normal but not close to the inverse Gaussian distribution. This is not consistent with stochastic rise to threshold models, and implies that between-trial rate (reciprocal RT) is a fundamental variable. There are significant between-trial temporal and spatial sequential dependencies extending back about 3 lags. When tasks become difficult, the rate distributions shift to the left and becomes truncated near zero. We deduced true truncation could not occur due the sequential dependency, but rate distributions are still close to the truncated Normal. Responding to back-to-back sequences of hundreds of almost identical RT trials is not a natural behavior. Nevertheless, it does reflect decision-making when there is time pressure. We propose that when gain in utility is an increasing concave function of time (speed-accuracy trade-off) there emerges an optimal time of response when time is a penalty. We propose that response rate reflects such a process and argue against the longstanding assumption of rise-to-threshold.

### Conflict of interest statement

The authors declare that the research was conducted in the absence of any commercial or financial relationships that could be construed as a potential conflict of interest.
